# Large-Scale Transcriptome Analysis Identified a Novel Cancer Driver Genes Signature for Predicting the Prognostic of Patients With Hepatocellular Carcinoma

**DOI:** 10.3389/fphar.2021.638622

**Published:** 2021-07-16

**Authors:** Gao Li, Xiaowei Du, Xiaoxiong Wu, Shen Wu, Yufei Zhang, Jing Xu, Hao Wang, Tingsong Chen

**Affiliations:** ^1^ Second Department of Oncology, Seventh People's Hospital of Shanghai University of Traditional Chinese Medicine, Shanghai, China; ^2^ Postgraduate College, Jinzhou Medical University, Jinzhou, China; ^3^ Department of Oncology, General Hospital of Northern Theater Command, Shenyang, China

**Keywords:** cancer driver genes, prognostic signature, hepatocellular carcinoma, TCGA, ICGC

## Abstract

**Background:** Hepatocellular carcinoma (HCC) is a common malignant tumor with high mortality and heterogeneity. Genetic mutations caused by driver genes are important contributors to the formation of the tumor microenvironment. The purpose of this study is to discuss the expression of cancer driver genes in tumor tissues and their clinical value in predicting the prognosis of HCC.

**Methods:** All data were sourced from The Cancer Genome Atlas (TCGA), International Cancer Genome Consortium (ICGC), and Gene Expression Omnibus (GEO) public databases. Differentially expressed and prognostic genes were screened by the expression distribution of the cancer driver genes and their relationship with survival. Candidate genes were subjected to functional enrichment and transcription factor regulatory network. We further constructed a prognostic signature and analyzed the survival outcomes and immune status between different risk groups.

**Results:** Most cancer driver genes are specifically expressed in cancer tissues. Driver genes may influence HCC progression through processes such as transcription, cell cycle, and T-cell receptor-related pathways. Patients in different risk groups had significant survival differences (*p* < 0.05), and risk scores showed high predictive efficacy (AUC>0.69). Besides, risk subgroups were also associated with multiple immune functions and immune cell content.

**Conclusion:** We confirmed the critical role of cancer driver genes in mediating HCC progression and the immune microenvironment. Risk subgroups contribute to the assessment of prognostic value in different patients and explain the heterogeneity of HCC.

## Background

Liver cancer is the sixth most common malignancy, with a high mortality rate and a five-year survival rate of less than 18 percent (Jemal et al., 2017; Villanueva, 2019; Siegel et al., 2020). Hepatocellular carcinoma (HCC) accounts for over 80 percent of liver cancer and is highly malignant, heterogeneity, and recurrent (Yi et al., 2018; Prince et al., 2020). Alpha-fetoprotein (AFP) is the most common biomarker for HCC, though with insufficient specificity. Approximately 31% of patients with HCC have AFP less than 400 μg/L, and it tends to decrease with age (Chen and Sung, 1977). Besides, patients with the same tumor stage sometimes had survival differences (Liu et al., 2019). In the last decade, studies have shown that different molecular typing influences the response to treatment and prognosis of HCC, such as the expression status of programmed cell death protein 1 (PD-1), suggesting different sensitivity to immunotherapy (Scheiner et al., 2019; Feng et al., 2020a). New molecular typing studies are expected to provide novel biomarkers with higher specificity and sensitivity for predicting the prognosis of HCC.

Tumorigenesis is often combined with alterations in the stromal environment, immune cells, and immune function, and is a complex multifactorial process (Principe et al., 2016; Ruhland et al., 2016). The journal of Nature Reviews Cancer recently reported changes at the molecular level during the malignant progression of tumors (Martínez-Jiménez et al., 2020). Their study produced the most comprehensive profile of cancer driver genes to date and proposed mechanisms of tumorigenesis. Through a holistic analysis of more than 20,000 samples from 66 tumors, 568 cancer driver genes were identified, and these molecules are considered to be the main triggers of genetic mutations and uncontrolled cell growth. Cancer driver genes affect various aspects of tumorigenesis and development (Bossi et al., 2016; Zhao et al., 2019), and research on cancer driver genes is an opportunity to advance targeted drug therapy against cancer and to find biomarkers for tumor prognosis and therapeutic response.

In this study, we analyzed the expression of 568 cancer driver genes in HCC, the possible regulatory networks, and functional pathways by which driver genes influence the malignant progression of HCC. We constructed a multi-gene risk signature to predict the prognosis of patients with different risk groups. Our study is the first to investigate the clinical value of cancer driver genes in HCC from a prognostic perspective, which is expected to become a novel biomarker for HCC and provide strategies for treatment and drug response.

## Materials and Methods

### Data Sources

Hepatocellular carcinoma transcriptome sequencing data and corresponding clinical data were acquired from The Cancer Genome Atlas (TCGA) (https://portal.gdc.cancer.gov/), International Cancer Genome Consortium (ICGC) (https://icgc.org/) and Gene Expression Omnibus (GEO) (https://www.ncbi.nlm.nih.gov/geo/) databases. The TCGA database comprises 424 tissues, with 374 cancerous tissues and 50 paracancerous tissues. The ICGC database contains 445 tissues, including 202 paracancerous tissues and 243 cancerous tissues. The pathological characteristics of the follow-up patients were as follows: overall survival (OS), disease-free survival (DFS), survival status, age, gender, tumor stage, grade, and the corresponding patient information is shown in Table 1. The TCGA and ICGC databases were used to screen risk cancer driver genes and construct the prognostic signature, and the GSE76427 set in the GEO was used as external validation of the signature.

**TABLE 1:** Clinical features of HCC patients from TCGA database and ICGC database.

Variables	TCGA (*n* = 365)		ICGC (*n* = 211)	
	N	%	N	%
Age, years	222	60.8	78	36.9
≤65	133	36.4	133	63.1
>65				
Gender	241	66.0	158	74.9
Male	114	31.2	53	25.1
Female				
Grade	53	14.5	20	9.5
G1	170	46.6	136	64.5
G2	116	31.8	55	26.0
G3	11	3.0	-	
G4				
Stage stage i	167	45.8	32	15.2
stage ii	80	21.9	102	48.3
stage iii	80	21.9	63	29.8
stage iv	4	1.1	14	6.7

There were 568 cancer driver genes obtained from the Integrative OncoGenomics platform (https://www.intogen.org/search). Expression profiles of these molecules were extracted from transcriptomic data and the level 3 RNA sequencing data from different platforms were normalized using the “limma” and “sva” R packages.

### Screening for Differentially Expressed and Prognostic Genes

We firstly analyzed the expression of cancer driver genes in tumor tissues and paracancerous tissues and filtered out differentially expressed genes (DEGs) (*p* < 0.05) using the “limma” R package. Similarly, we used univariate Cox analysis to screen for prognostic genes (PGs) associated with overall survival (OS) (*p* < 0.05) by the “survival”R package. Venn diagrams were drawn to analyze DEGs and PGs in TCGA and ICGC cohort. These cancer driver genes are considered to be potentially involved in the malignant progression of HCC and are associated with patient survival outcomes.

### Construction of Transcription Factor Regulatory Networks and Functional Enrichment Analysis

Next, we performed functional enrichment analysis of the screened cancer driver genes. Acquisition of 318 cancer-associated transcription factors at the Cistrome platform (http://cistrome.org/). Cytoscape software was used to map the regulatory network between the transcription factors that are highly correlated (|*R* ^2^| > 0.5 and *p* < 0.05) with the cancer driver genes. Besides, we also performed Gene Ontology (GO) and Kyoto Encyclopedia of Genes and Genomes (KEGG) enrich analysis using the “enrichplot” R package, which suggested the regulatory mechanisms and pathways by which cancer driver genes affect the prognosis of HCC.

### Construction and Validation of the Risk Signature

To further confirm the correlation between cancer driver genes and survival outcomes of HCC patients, we constructed a risk signature using Least absolute shrinkage and selection operator (Lasso) Cox regression and stepwise multivariate Cox proportional regression. The TCGA cohort was the training set, while the ICGC and GEO cohort were the validation sets. According to the median risk score of the training set, all patients were categorized into high-risk and low-risk groups, and survival curves were used to compared OS or DFS differences among risk groups. Principal component analysis (PCA), t-distributed stochastic neighbor embedding (t-SNE), and receiver operating characteristic (ROC) curves to validate the risk signature’s specificity and sensitivity. Univariate and multivariate regression were used to analyze the independent value of risk scores for predicting HCC prognosis.

### Correlation Analysis of Immune Status

We also tried to investigate the correlation between immune cells and immune function with cancer driver genes risk signature. Immunological parameters were scored for TCGA and ICGC cohort cases using the single-sample gene set enrichment analysis (ssGSEA) algorithm through the “gsva” R package, including 16 immune cell scores and 13 immune-related pathway scores. Correlation analysis of immune indices with different risk groups suggests possible ways in which cancer driver genes affect immune status.

### Statistical Analysis

The statistical analyses were performed with R v3.4.1 (https://www.r-project.org/). Expression differences in mRNA transcriptome were tested by the Wilcoxon method. The correlation between cancer driver genes and transcription factors was analyzed by Pearson’s correlation coefficient method. Kaplan-Meier method was used for survival analysis. Mann–Whitney U test was used to compare immune cell scores or immune-related pathway scores between the high-risk and low-risk groups. The independent prognostic value was performed with univariate and multivariate regression analysis.

## Results

### Differentially Expressed and Prognostic Genes in TCGA and ICGC Cohort

**FIGURE 1:**
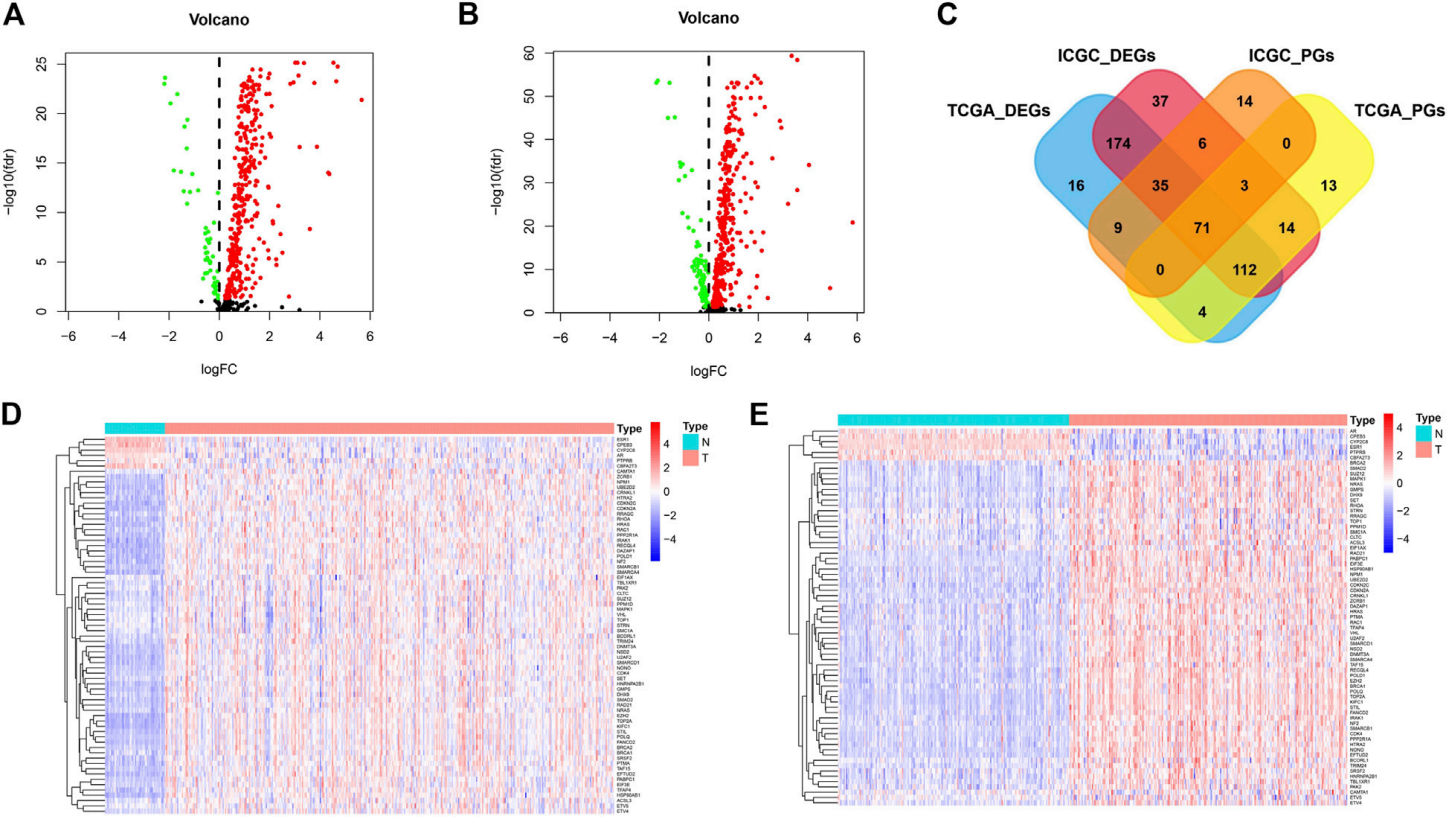
The differentially expressed and prognostic genes in TCGA cohort and ICGC cohort. **(A)** The expression of cancer driver genes between tumor tissues and paracancerous tissues in TCGA cohort. **(B)** The expression of cancer driver genes between tumor tissues and paracancerous tissues in ICGC cohort. **(C)** Venn diagrams for differentially expressed and prognostic genes. **(D)** The expression distribution of screened cancer driver genes in TCGA cohort. **(E)** The expression distribution of screened cancer driver genes in ICGC cohort.

Among the 568 cancer driver genes, 371 genes were higher expressed in tumor tissues than that in paracancerous tissues (*p* < 0.05), and 50 genes were lower expressed in paracancerous tissues in the TCGA cohort (*p* < 0.05) (Figure 1A). In the ICGC cohort, 369 genes were expressed higher in the tumor tissues than paracancerous tissues (*p* < 0.05), and 83 genes were lower expressed in paracancerous tissues (*p* < 0.05) (Figure 1B). Most of the differential genes in both cohorts are consistent, and these cancer driver genes may be involved in driving mutations in cancer and promoting malignant tumor progression.

We also further analyzed cancer driver genes associated with survival time in HCC patients. In the TCGA cohort, 202 molecules were risk factors (HR > 1 and *p* < 0.05) and 15 molecules were protective factors (HR < 1 and *p* < 0.05) affecting patient survival. In the ICGC cohort, 87 molecules were risk factors (HR > 1 and *p* < 0.05) and 51 molecules were protective factors (HR < 1 and *p* < 0.05) affecting patient survival. We plotted Venn diagrams for a total of 71 molecules whose expression was both a differential gene and a prognostic gene (Figure 1C). The expression distribution of these screened cancer driver genes in tumor tissues and paracancerous tissues is shown in Figures 1D,E. For these candidate molecules, our further step is to perform functional enrichment analysis and transcriptional regulatory network construction.

### Transcription Factor Regulatory Networks and Functional Enrichment Analysis

**FIGURE 2:**
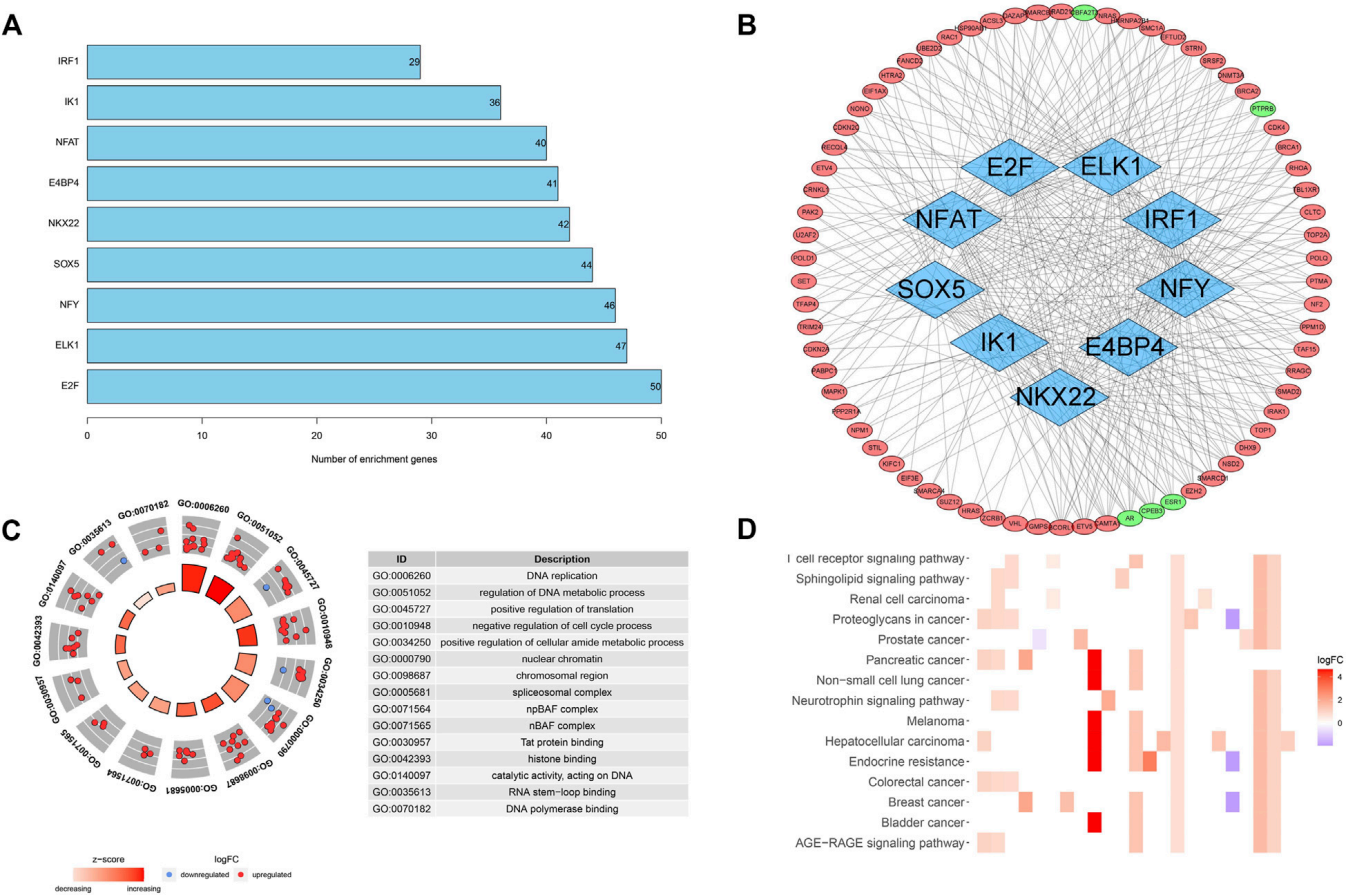
Transcription factor regulatory networks and functional enrichment analysis. **(A)** The number of cancer driver genes that are highly correlated with transcription factors. **(B)** Regulatory networks of transcription factors and cancer driver genes. **(C)** GO analysis of screened cancer driver genes. **(D)** KEGG analysis of screened cancer driver genes.

Pearson’s correlation coefficient method validated nine transcription factors highly correlated (|*R* ^2^| > 0.5 and *p* < 0.05) with screened cancer driver genes, all of which were enriched for more than 20 genes (Figure 2A). We plotted the regulatory networks of these transcription factors against screened cancer driver genes, of which AR, CPEB3, ESR1, PTPRB, and CBFA2T3 are lower expressed in cancer tissues compared with the paracancerous tissues (*p* < 0.05), and the others are higher expressed in cancer tissues (*p* < 0.05) (Figure 2B). The regulatory network reveals key nodes where cancer driver genes are regulated by the transcriptome, contributing to inform further research on the relationship between transcription and driver mutations.

We also analyzed the function and pathway enrichment of screened cancer driver genes. GO analysis suggested a significant enrichment of biological processes such as DNA replication and metabolism, and cell cycle (Figure 2C). KEGG analysis suggested that cancer driver genes may function through hepatocellular carcinoma, AGE-RAGE signaling pathway, and T-cell receptor signaling pathway (Figure 2D). These results also validate the close linkage between driver genes and transcriptional processes, which is also beneficial to the study of the mechanisms by which driver genes affect tumorigenesis.

### Construction of the Four-mRNA Signature for Predicting Patient Prognosis

**FIGURE 3:**
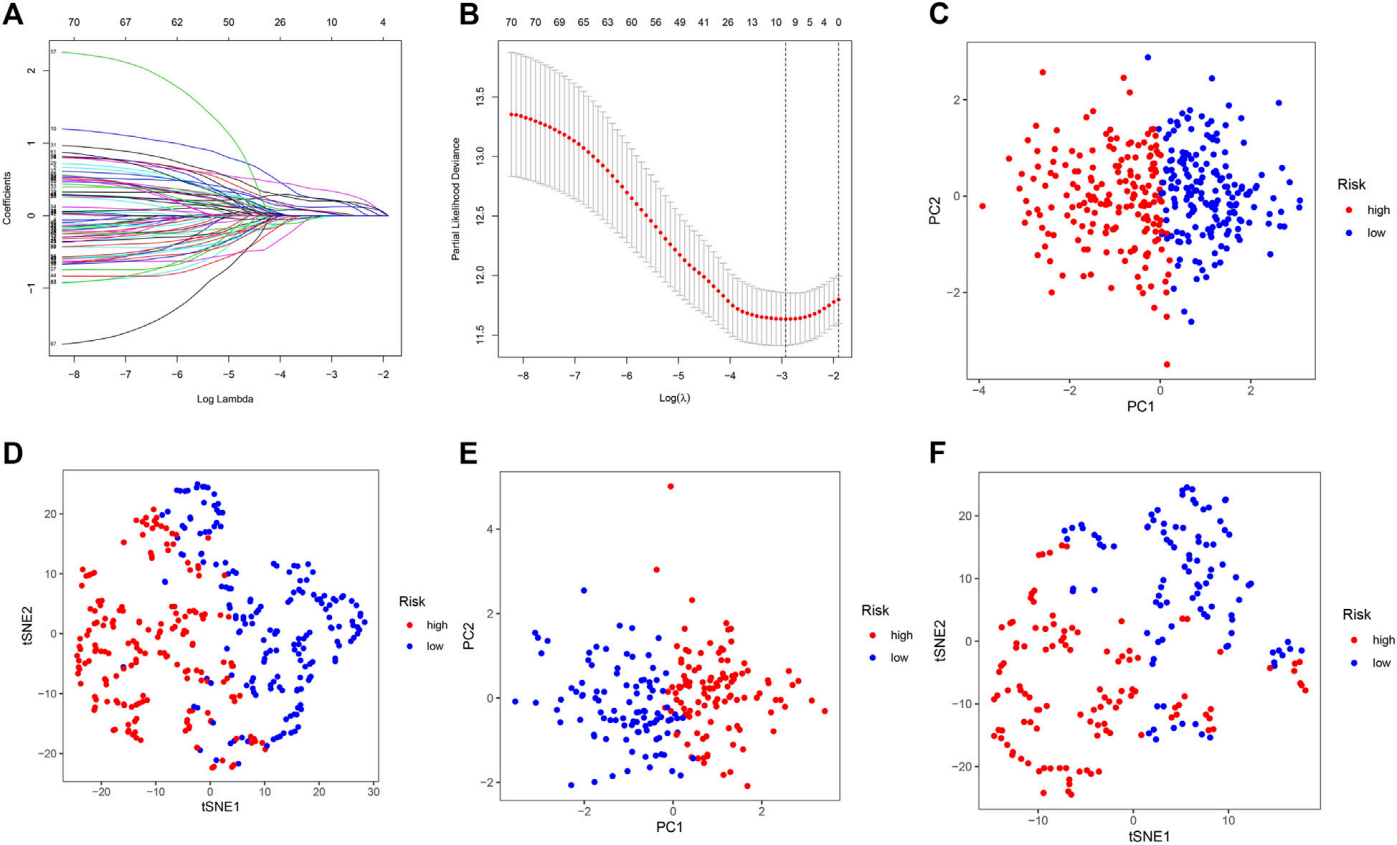
Construction and validation of the cancer driver genes signature in the TCGA and ICGC cohort. **(A–B)** Lasso regression screens candidate risk cancer driver genes. **(C)** PCA validation of the distribution of different risk groups in the training set. **(D)** T-SNE validation of the distribution of different risk groups in the training set. **(E)** PCA validation of the distribution of different risk groups in the ICGC set. **(F)** T-SNE validation of the distribution of different risk groups in the ICGC set.

Based on the expression of 71 screened cancer driver genes and combining OS in the TCGA cohort, we performed the first step of screening for risk signature genes. We obtained a total of nine candidate risk molecules through Lasso regression (Figures 3A,B). We further performed a stepwise multivariate Cox regression to complete the signature construction, and finally screened out four signature risk molecules (Table 2). The risk score for each patient could be calculated using the following formula, risk score = e^(expression of ETV5*0.338+expression of EZH2*0.436+expression of PABPC1*0.234+expression of ZCRB1 *0.692)^. Depending on the median risk score, all patients were categorized into high-risk and low-risk groups. Similarly, we calculated risk scores for all patients in the ICGC cohort using the risk signature formula, and all patients were categorized into high-risk and low-risk groups.

**TABLE 2:** Each gene coefficients of the four-mRNA signature.

mRNA	Coefficient	HR	95%CI	P
ETV5	0.338	1.403	1.077–1.827	0.012
EZH2	0.436	1.547	1.124–2.130	0.007
PABPC1	0.234	1.264	0.994–1.606	0.056
ZCRB1	0.692	1.998	1.235–3.232	0.005

We used PCA and t-SNE analyses to assess the signature fit. The different risk groups showed a two-way distribution, which indicated that the risk score could distinguish the distribution patterns of the different risk groups (Figures 3C–F). The results of the training set were consistent with those of the test set, suggesting that the signature we constructed could better distinguish between groups with different expressions of cancer driver genes.

### Validation of the Four-mRNA Signature in Predicting Patient Prognosis

**FIGURE 4:**
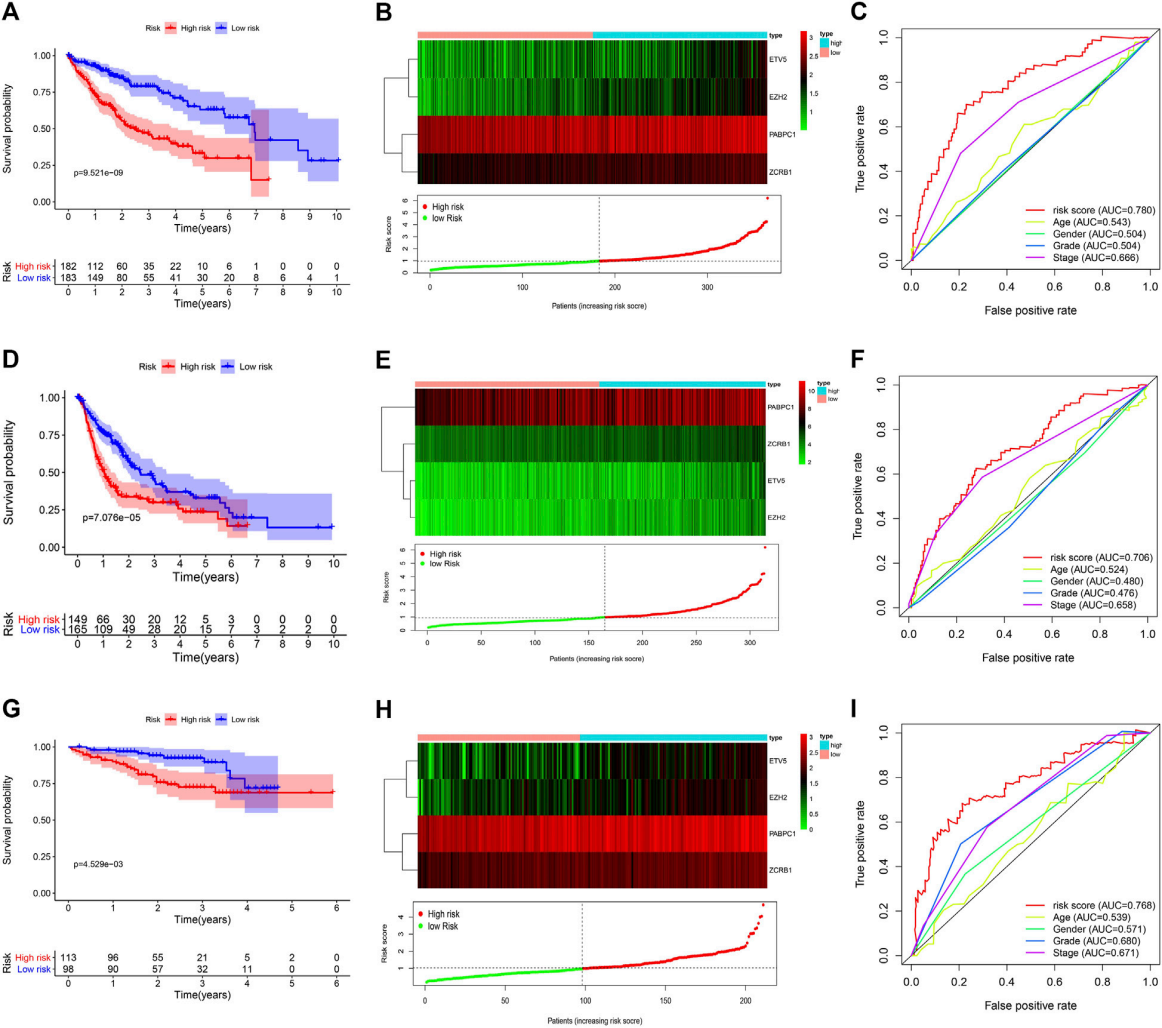
Survival analysis and sensitivity validation of the risk signature in the TCGA and ICGC cohort. **(A)** OS analysis of different risk groups in the TCGA cohort. **(B)** The expression distribution of risk genes in TCGA cohort. **(C)** The ROC curve validated the sensitivity and specificity of the risk score to predict patient OS in TCGA cohort. **(D)** DFS analysis of different risk groups in the TCGA cohort. **(E)** The expression distribution of risk genes in TCGA cohort. **(F)** The ROC curve validated the sensitivity and specificity of the risk score to predict patient DFS in TCGA cohort. **(G)** OS analysis of different risk groups in the ICGC cohort. **(H)** The expression distribution of risk genes in ICGC cohort. **(I)** The ROC curve validated the sensitivity and specificity of the risk score to predict patient OS in ICGC cohort.

We explored the early warning effect of the risk signature on clinical prognosis. In the TCGA cohort, survival analysis suggested that the overall survival rate of the low-risk group was higher than that of the high-risk group (*p* < 0.001) (Figure 4A), and the expression of candidate risk molecules in different risk groups is shown in Figure 4B. The ROC curves validate the sensitivity and specificity of the risk signature, and the risk score showed a higher predictive effect compared with other clinicopathological characteristics (Figure 4C). We also use cancer-specific disease-free survival as an endpoint for evaluating the prognostic value of the risk signature. The results also suggested a higher disease-free survival rate in the low-risk group compared with the high-risk group (*p* < 0.001), and risk scores performed high sensitivity and specificity (AUC:0.706) (Figures 4D–F). Further, we used the ICGC cohort for validation of the signature, and the results were consistent with the TCGA set. Patients in the high-risk group had lower survival rates (*p* < 0.01) (Figures 4G,H) and the predictive performance of the risk score was quite effective (Figure 4I).

**FIGURE 5:**
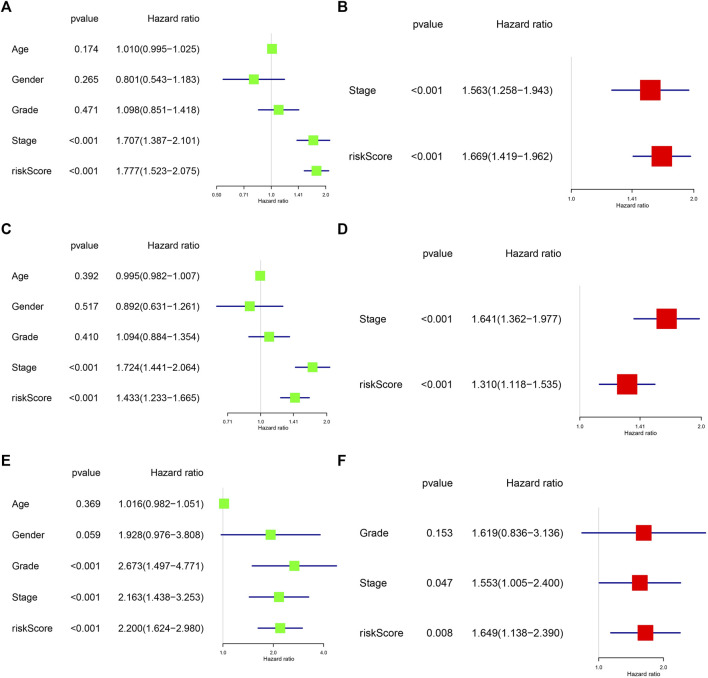
Analysis of the independent prognostic value of risk scores in HCC. **(A)** Univariate regression analysis of clinical characteristics and risk score affecting patient OS in the TCGA cohort. **(B)** Multivariate regression analysis of clinical characteristics and risk score affecting patient OS in the TCGA cohort. **(C)** Univariate regression analysis of clinical characteristics and risk score affecting patient DFS in the TCGA cohort. **(D)** Multivariate regression analysis of clinical characteristics and risk score affecting patient DFS in the TCGA cohort. **(E)** Univariate regression analysis of clinical characteristics and risk score affecting patient OS in the ICGC cohort. **(F)** Multivariate regression analysis of clinical characteristics and risk score affecting patient OS in the ICGC cohort.

We also validated the independent prognostic role of the risk signature using univariate and multivariate regression analysis. In the TCGA cohort, univariate regression analysis suggested that stage and risk score were significantly associated with patient’s OS and DFS (*p* < 0.001). After adjusting for other interfering factors, multivariate regression analysis suggested that risk score could be an independent risk factor affecting OS and DFS of HCC patients (HR > 1 and *p* < 0.001) (Figures 5A– D). In the ICGC validation cohort, we also confirmed the independent prognostic value of the risk score in HCC patients (HR > 1 and *p* < 0.01) (Figures 5E,F). Those results suggest the important role of the risk signature in predicting the prognosis of HCC patients.

### External Validation of the Four-mRNA Signature in GEO Cohort

**FIGURE 6:**
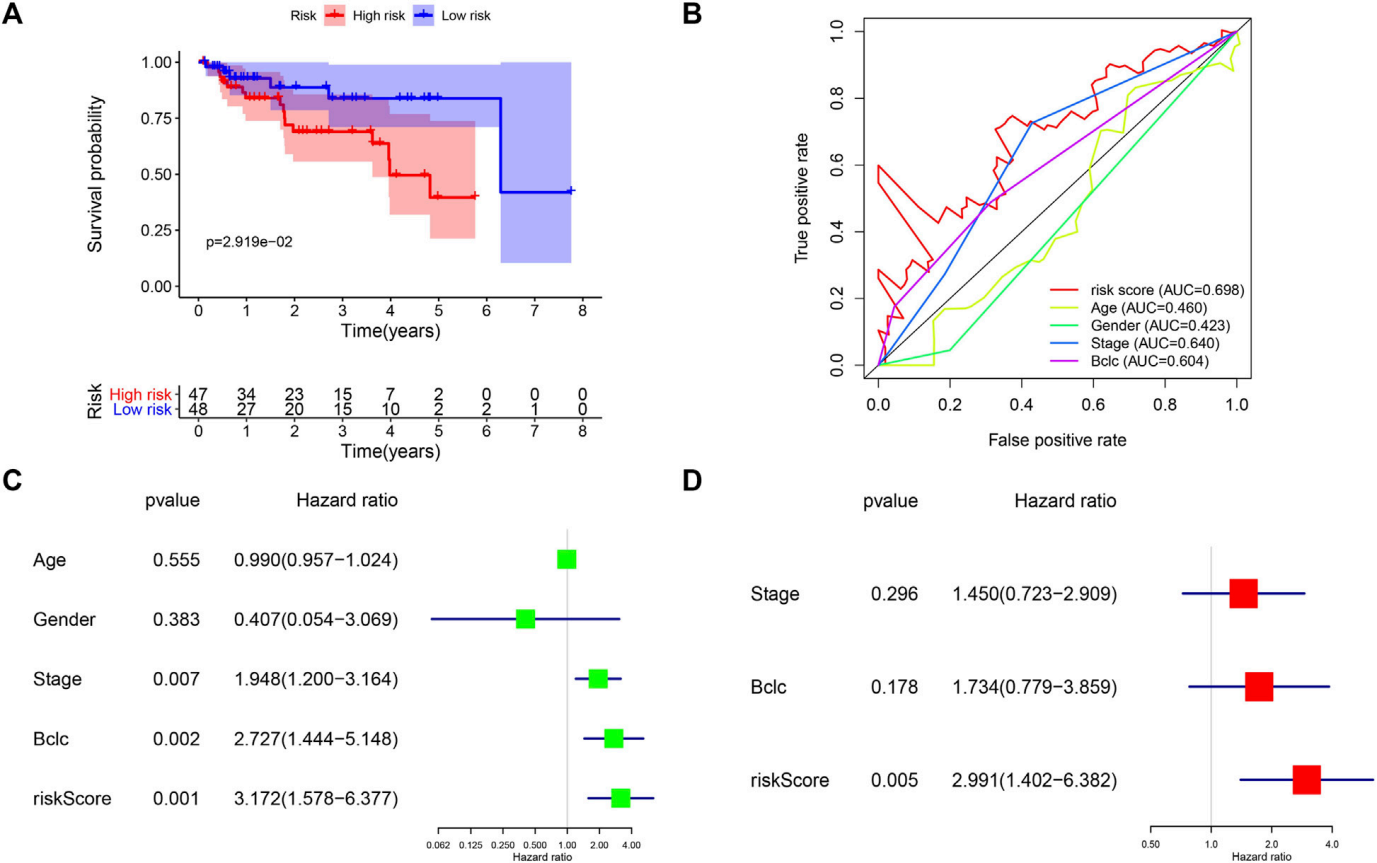
External validation of the four-mRNA signature in GEO cohort. **(A)** Survival analysis of different risk groups in the GEO cohort. **(B)** ROC curves validate the sensitivity and specificity of the risk model in the training set. **(C)** Univariate regression analysis of clinical characteristics and risk score affecting patient OS in the GEO cohort. **(D)** Multivariate regression analysis of clinical characteristics and risk score affecting patient OS in the GEO cohort.

TCGA and GEO cohorts are used to screen risk molecules and construct the risk signature, and external validation of the GSE76427 set can evidence the high specificity and sensitivity of the signature. We calculated the risk score for each patient using the risk score formula, and all patients were categorized into high-risk and low-risk groups according to the median risk score of the training set. Survival curves indicated that patients in the high-risk group had lower survival rates than those in the low-risk group (*p* < 0.05) (Figure 6A). The AUC values of the risk scores were higher than other clinicopathological characteristics (Figure 6B). Univariate and multivariate regression suggest that risk score was an independent risk factor affecting the prognosis of HCC (HR > 1 and *p* < 0.01) (Figures 6C,D). The results of the external validation similarly approved the applicability of the risk score, and the cancer driver gene signature has great potential as a novel biomarker for HCC.

### Correlation of the Risk Signature With Immune Status

**FIGURE 7:**
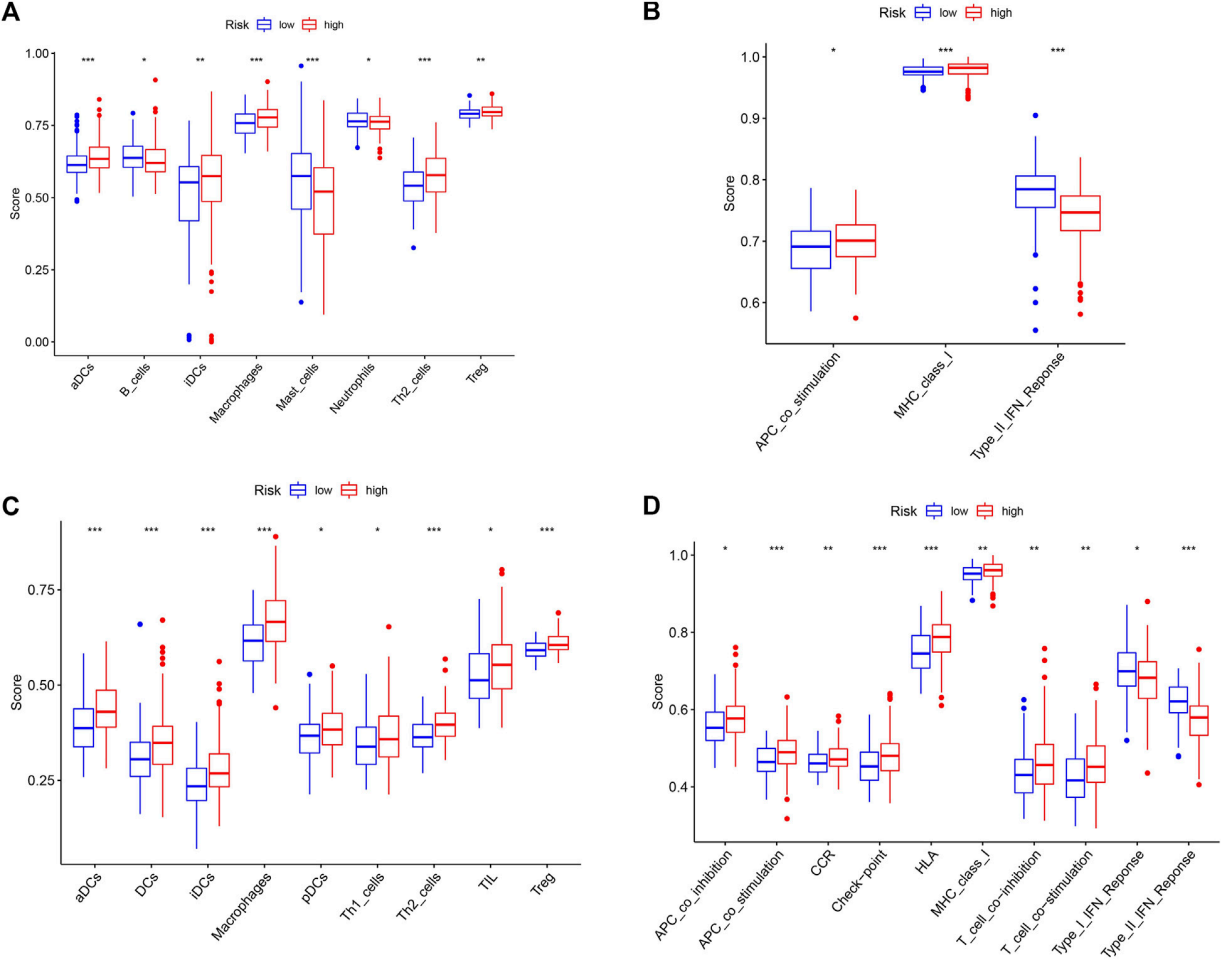
Correlation analysis of risk groups and immune status. **(A)** Immune cell scores for different risk groups in the TCGA cohort. **(B)** Immune-related pathway scores for different risk groups in the TCGA cohort. **(C)** Immune cell scores for different risk groups in the ICGC cohort. **(D)** Immune-related pathway scores for different risk groups in the ICGC cohort. *, *p* < 0.05; **, *p* < 0.01; ***, *p* < 0.001.

KEGG suggests the association of cancer driver genes with T cell-related pathways, and we were equally curious about the correlation between risk signature and immune status. In the TCGA cohort, the score of activated dendritic cell (aDC), immature dendritic cell (iDC), Macrophages, Th2 cells, and Treg immune cells was higher in the high-risk group than that in the low-risk group (*p* < 0.05), and the score of B cells, Mast cells, and Neutrophils immune cells were lower in the high-risk group compared with the low-risk group (*p* < 0.05) (Figure 7A). The score of antigen-presenting cell (APC) co_stimulation, major histocompatibility complex (MHC) class I, type II interferon (IFN) response immune function was associated with risk groups (*p* < 0.05) (Figure 7B). In the ICGC cohort, risk grouping was also associated with cytokine-cytokine receptor (CCR), checkpoint, human leukocyte antigen (HLA), T cell co-inhibition, T cell co-stimulation immune function, and DCs, plasmacytoid dendritic cell (pDC), Th1 cells, tumor infiltrating lymphocyte (TIL) immune cell content correlated (*p* < 0.05) (Figures 7C,D). These results suggest that the immune status of patients in different risk groups differs and that immune function is equally important for patient prognosis.

## Discussion

In recent decades, the treatment of HCC has entered the age of precision-targeted and immunotherapy, where multiple drugs targeting vascular receptors or immune checkpoints have greatly increased the survival benefits for patients (Nguyen et al., 2015; Chacko and Samanta, 2016). However, biomarker updates for cancer drug response or prognosis have not kept pace. Traditional tumor staging and tumor markers such as AFP and carcinoembryonic antigen (CEA) are still the most common indicators of tumor prognosis and treatment (Pang et al., 2015). With the advent of second-generation sequencing and the establishment of biospecimen repositories around the world, new molecular typing studies for tumor prediction are on the rise (Joly et al., 2012; Tomczak et al., 2015). For example, Hu et al. (2020) studied the immune features of the tumor microenvironment. They constructed an immune-related gene signature and multiple immune subgroups for HCC prognosis, which contribute to explain the high heterogeneity of HCC and provide a reference for individualized treatment. RNA methylation and long noncoding RNA (lncRNA) are important elements in the regulation of transcriptome expression. Wu et al. (2020) study constructed an RNA methylation signature that predicts the prognosis of HCC patients with high precision. Lin et al. (2020) studied the methylation levels of lncRNA initiation sites to explore different methylation site alterations that affect the survival of HCC patients. Other studies, such as autophagy-related signatures and ferroptosis-related signatures (Wang et al., 2020a; Liang et al., 2020), are also increasing. Among these studies, many new molecular typing studies performed high specificity and predictive validity, and they have shown great promise for clinical application. Driver genes that promote cancer mutations are a major contributor to tumor malignancy, and studies on the prognostic signature of HCC driver genes are currently lacking.

A primary feature of cancer is the altered microenvironment caused by cancer mutations (Hanahan and Weinberg, 2000; Mumenthaler et al., 2015). Through the study of mutation data from a large number of tumor specimens, Francisco et al. (Martínez-Jiménez et al., 2020) identified 568 driver genes that promote cancer mutations, which is one of the most comprehensive studies of cancer driver genes to date. Conway et al. (2020) have integrated relevant genes that drive the biological behavior of melanoma, and they have also identified a few key DNA repair genes that can be used to clinically subgroup patients who may benefit from currently unconsidered therapeutic modalities.

Cancer driver genes may also have much to teach us about solid tumors. In our study, we found that most cancer driver genes are specifically expressed in tumor tissue, and we constructed a four-mRNA prognostic signature (ETV5, EZH2, PABPC1, ZCRB1) that has higher predictive power than other clinical features. Related studies have also found that these molecules are associated with multiple processes in tumor development. ETV5 may promote malignant progression of thyroid cancer through the PIK3CA signaling pathway (Meng et al., 2020), and ETV5 expression has also been associated with resistance to treatment with bevacizumab in colorectal cancer (Feng et al., 2020b). EZH2 affects the prognosis of breast cancer patients and the choice of EZH2 inhibitory drugs for ovarian cancer (Djordjevic et al., 2020; Leitner et al., 2020). PABPC1 can be used to predict the clinical outcome of glioma patients (Wang et al., 2020b). However, ZCRB1 has been less studied in cancer. Their study demonstrates the important role of cancer driver genes in cancer and is a great support to our results. Overall, our study presents a novel cancer driver gene-related signature for predicting HCC prognosis, and the new signature exhibits an independent predictive role and high predictive performance.

Immune cell alterations and specific matrix are one of the essential pillars in the construction of the tumor microenvironment and the initial motive for the promotion of unlimited tumor cell multiplication and invasion into other tissues (Sun et al., 2013; Beachley et al., 2015). Our study also demonstrates the involvement of these immune functional assemblies and immune cells in the process of tumor mutagenesis, which may indirectly affect the survival prognosis of patients. Immune cell alterations such as dendritic cells and Th2 cells are important triggers of tumor immune infiltration and subsequent vascular invasion, and their activation also correlates with the patient’s response to immune and biological vaccine therapy (Moreira et al., 2020; Patel et al., 2020; Zhao et al., 2020). HLA and CCR also affect various aspects of the body’s immune system (Attia et al., 2020). HLA and CCR in progressive tumors are more likely to be continuously activated and maintain the complex tumor microenvironment (Pankratova et al., 2016). Although the process of tumorigenesis is complex, understanding their relationship to driver genes and immune status will help us to better sequence therapy and assess patient clinical outcomes.

## Conclusion

In summary, cancer driver genes are closely associated with the malignant progression and clinical prognosis of HCC. Our study identified several cancer driver genes involved in multiple biological behaviors of tumors and analyzed the regulatory networks and functional pathways they may mediate. We also constructed a predictive signature for patients’ prognosis, which showed high specificity and sensitivity. Our study of immune status also revealed their high correlation with cancer driver genes, providing a reference for the expansion of immunotherapy strategies and deeper mechanistic studies.

## Data Availability

Publicly available datasets were analyzed in this study. This data can be found here: All data was obtained from The Cancer Genome Atlas (TCGA) (https://portal.gdc.cancer.gov/), International Cancer Genome Consortium (ICGC) (https://icgc.org/) and Gene Expression Omnibus (GEO) (https://www.ncbi.nlm.nih.gov/geo/) databases.

## Abbreviations

aDC, Activated dendritic cell; AFP, Alpha fetoprotein; APC, Antigen-presenting cell; AUC, Area under the curve; CCR, Cytokine-cytokine receptor; CEA, Carcinoembryonic antigen; DEGs, Differentially expressed genes; GEO, Gene Expression Omnibus; GO, Gene Ontology; HCC, Hepatocellular carcinoma; HLA, Human leukocyte antigen; HR, Hazard ratio; ICGC, International Cancer Genome Consortium; iDC, Immature dendritic cell; IFN, Interferon; KEGG, Kyoto Encyclopedia of Genes and Genomes; LASSO, Least absolute shrinkage and selection operator; MHC, Major histocompatibility complex class I; OS, Overall survival; PCA, Principal component analysis; PD-1, Programmed cell death protein 1; pDC, Plasmacytoid dendritic cell; PGs, Prognostic genes; ROC, Receiver operating characteristic; TCGA, The Cancer Genome Atlas; TIL, Tumor-infiltrating Lymphocyte; t-SNE, T-distributed stochastic neighbor embedding.
